# ^18^F‐FDG PET/CT imaging of **s**mall intestinal metastasis from pulmonary sarcomatoid carcinoma: Brief report and review of the literature

**DOI:** 10.1111/1759-7714.13468

**Published:** 2020-05-15

**Authors:** Xinli Xie, Ning Tu, Qi Wang, Zhen Cheng, Xingmin Han, Lihong Bu

**Affiliations:** ^1^ Department of Nuclear Medicine the First Affiliated Hospital of Zhengzhou University Zhengzhou China; ^2^ PET‐CT/MRI Center & Molecular Imaging Center Wuhan University Renmin Hospital Wuhan China; ^3^ The 1^st^ Department of Gastrointestinal Surgery Wuhan University Renmin Hospital Wuhan China; ^4^ Molecular Imaging Program at Stanford (MIPS), Department of Radiology and Bio‐X Program Stanford University Stanford California USA

**Keywords:** ^18^F‐Fluorodeoxyglucose, non‐small cell lung cancer, positron emissiontomography/computed tomography, pulmonary sarcomatoid carcinoma, small intestinal metastasis

## Abstract

We herein report two cases of small intestinal metastasis from pulmonary sarcomatoid carcinoma (PSC) detected by ^18^F‐fluorodeoxyglucose positron emission tomography/computed tomography (^18^F‐FDG PET/CT). We reviewed the literature on ^18^F‐FDG PET/CT features in gastrointestinal metastasis of PSC patients since 1992, and further analyzed the imaging features. According to the literature review, 23 eligible cases were identified from eight studies, and no cases underwent ^18^F‐FDG PET/CT imaging. In this study, clinical and PET/CT imaging data of two patients are reported. In our cases, clinical and the CT images of lung masses were not typical, but the uptake of ^18^F‐FDG was remarkably high, with SUVmax exceeding 30. Small intestinal metastases may not be related to obstruction, or even the local intestinal cavity may be dilated. Therefore, in PSC patients with mild or without abdominal symptoms, ^18^F‐FDG PET/CT imaging could identify intestinal metastasis at a relatively early stage and may be used to determine the preferred biopsy site, or early intervention by surgery.

**Key points:**

^18^F‐FDG PET/CT imaging of small intestinal metastasis of PSC has not been previously reported in the literature and here we report the ^18^F‐FDG PET/CT features of two cases.

The uptake of ^18^F‐FDG was remarkably high in both the primary tumor and metastatic intestinal lesion. ^18^F‐FDG PET/CT imaging may therefore be used to determine the preferred biopsy site or early intervention by surgery.

## Introduction

Pulmonary sarcomatoid carcinoma (PSC) is an infrequent, poorly differentiated, highly‐metastatic subset of non‐small cell lung cancer (NSCLC), characterized by coexistent sarcomatoid and epithelial squamous cell carcinoma components, with a reported incidence rate of about 0.4% of all pulmonary malignancies.^1^ PSC has a tendency to spread to various sites, but rarely metastasizes to the gastrointestinal system. Gastrointestinal metastasis of PSC can be asymptomatic at an early stage,^2^ but lethal when diagnosed at a late stage with a high risk of serious complications, such as intussusception, perforation, ileus, bleeding, and acute appendicitis, which require emergency surgical intervention.^3^, ^4^ ^18^F‐fluorodeoxyglucose positron emission tomography/computed tomography (^18^F‐FDG PET/CT) is indispensable in the preoperative staging of NSCLC before surgery, but until now, few cases of gastrointestinal metastasis from PSC have been reported.^5^, ^6^, ^7^, ^8^, ^9^, ^10^, ^11^, ^12^ No studies have recently reported the PET/CT features of gastrointestinal metastasis from PSC. We herein report two cases of small intestinal metastasis from PSC detected by ^18^F‐FDG PET/CT.

## Methods

Physical examination, laboratory analysis, ^18^F‐FDG PET/CT, diagnostic transbronchial lung biopsy, gastroscopy and biopsy were performed. Both patients underwent a Biograph TruePoint 64 PET/CT Scan (Siemens AG, Munich, Germany). The ^18^F was produced in Sumitomo HM‐20 cyclotron system (Sumitomo Heavy Industries. Ltd., Japan). The ^18^F‐FDG was automatically synthesized in a chemical synthesis module (Sumitomo Heavy Industries. Ltd., Japan) with a radiochemical purity >95%. After fasting for more than six hours in a calm state, the patients were intravenously injected with 0.12 mCi/kg of ^18^F‐FDG which was followed by lying in a dark room for approximately one hour. PET and nonenhanced CT imaging was performed after the patients emptied their bladders. Scanning was performed from the middle femur to the cranial vault. The PET images were reconstructed according to an iterative ordered subset expectation maximization method. The CT scan data were collected with 120 kV (with the current adjusted to the patient's bodyweight) and a gantry rotation speed of 0.8 seconds. The reconstruction thicknesses of the CT images were 3.27 mm, and the PET and CT images were individually transferred to Syngo MMWP (Siemens) workstations, respectively, to display frame‐on‐frame fusion images.

We then performed a comprehensive MEDLINE, PubMed and EMBASE search to identify all reported cases of gastrointestinal metastases of PSC since 1992 when the pathological subtype PSC was named, especially cases with ^18^F‐Fluorodeoxyglucose and positron emission tomography/computed tomography (^18^F‐FDG PET/CT). Keywords used included gastrointestinal metastases and pulmonary sarcomatoid carcinoma.

## Results

### Case 1

Case 1 was a 55‐year‐old male heavy smoker with a history of epigastric pain and melena for about one month. Physical examination showed conjunctival pallor but no palpable mass. Laboratory analysis demonstrated anemia (Hct 24.9%) and leukocytosis (WBC 10.6 × 10^9^/L), while other laboratory results were almost normal, including electrolytes, blood sugar, tumor markers, liver and renal function. A previous CT scan from another hospital had demonstrated a mass in the left upper lobe.

A gastroscopy was performed to investigate the cause of melena and anemia, which detected erosive gastritis, dodecadactylitis and mild esophagitis. PET/CT images revealed a peripheral mass with intensely increased ^18^F‐FDG uptake in the left upper lobe measuring 3.7 × 3.3 × 3.1 cm. The maximum standardized uptake value (SUVmax) was calculated to be 33.2, which indicated a considerable high possibility of malignancy. The CT images of the lung mass showed both carcinomatous features including lobulation, irregular cavity, spiculation and pleural retraction, and sarcomatoid features including increased size, obvious necrosis unmatched with size, pleural involvement, and cavitation within the tumor. The mediastinal lymph node next to the aortic arch was mildly enlarged, measuring 1.5 × 0.9 cm with a SUVmax of 3.4. It is unusual for an enlarged lymph node to be palpated in the area beside the aortic arch and several mediastinal lymph nodes with this metabolic pattern have been proven by surgery to be metastasic in clinical practice, and this lymph node was therefore considered as suspect mediastinal lymph node metastasis.

**Figure 1:**
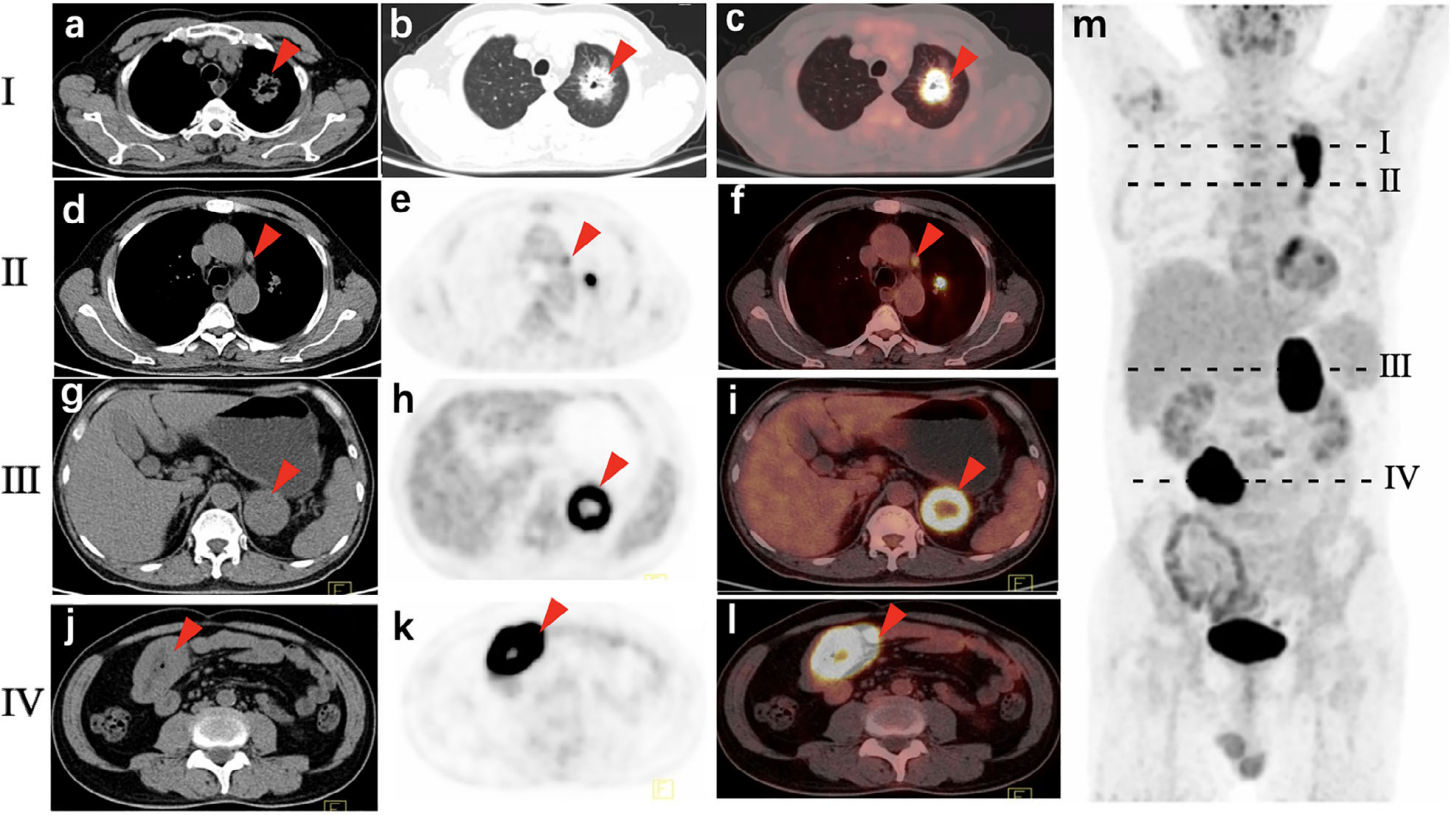
^18^F‐FDG PET/CT images of Case 1. **I**. (**a**) Soft tissue window; (**b**) lung window; and (**c**) PET/CT fusion image of left lung lesion (arrow) showed an irregular mass with lobulation, burr, central cavity and annular marked FDG uptake with maximum standardized uptake value (SUVmax) of 33.2. **II**. (**d**) Transaxial CT; (**e**) PET; and (**f**) PET/CT fusion image of a mediastinal lymph node measuring 1.5 × 0.9 cm with a SUVmax of 3.4. **III**. (**g**) Transaxial CT; (**h**) PET; and (**i**) PET/CT fusion image of left adrenal gland lesion showed a round mass measuring 7.6 × 5.1 × 5.0 cm, characterized by obvious necrosis with remarkably high FDG uptake (SUVmax:30.8). **IV**. (**j**) Transaxial CT; (**k**) PET; and (**l**) PET/CT fusion image of the intestinal lesion showed thickening intestinal wall up to 2.5 cm thickness and 7.3 cm long, with SUVmax of 41.6 in the right middle abdomen. No intestinal stenosis was observed. (**m**) Maximum intensity projection image of whole body showed high uptake lesions (red arrow) in (**I**) left thorax; (**II**) mediastinal lymph node; (**III**) left adrenal area; and (**IV**) right middle abdomen.

**Figure 2:**
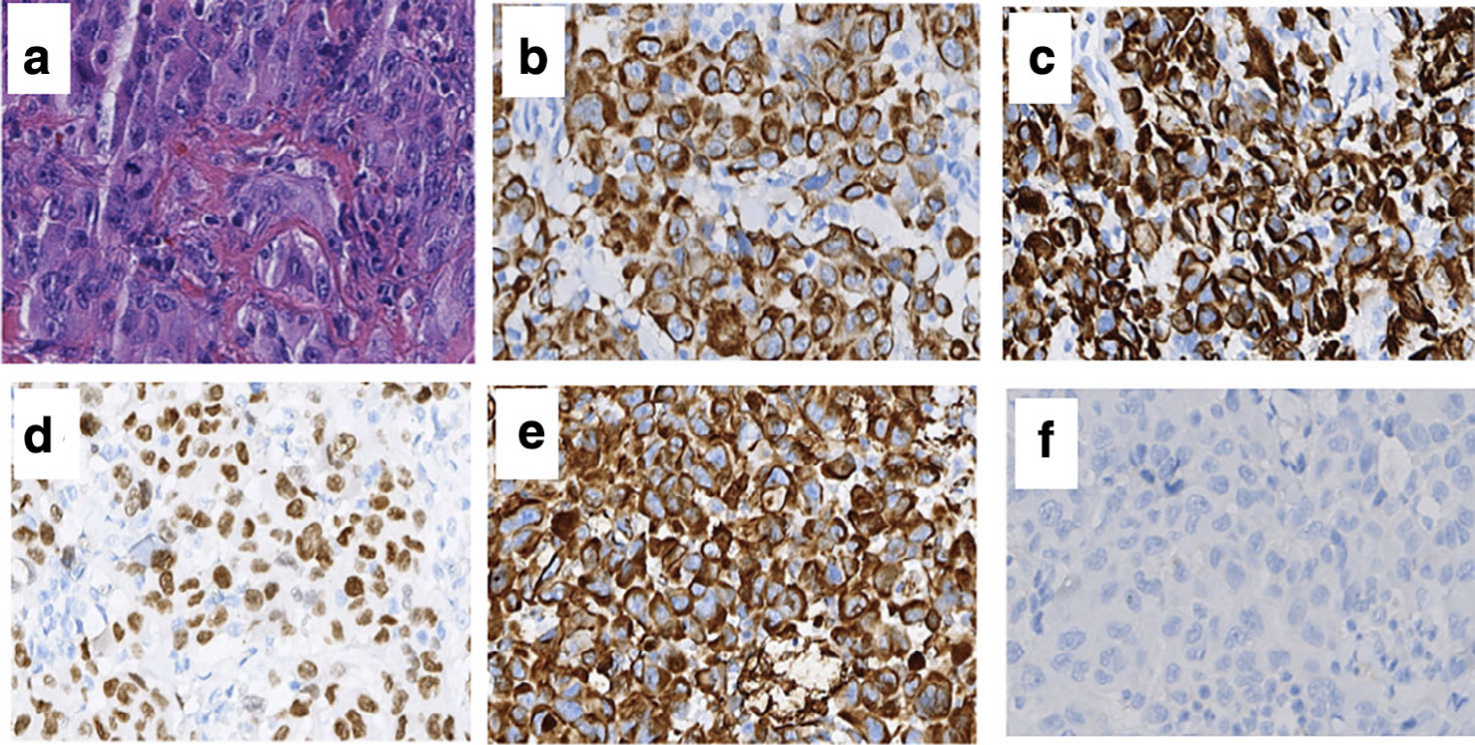
Hematoxylin and eosin stain (HE) and immunohistochemical staining of intestinal mass of Case 1 (×200). (**a**) HE staining; and (**b**–**f**) IHC markers staining showed CK (AE1/AE3) (+), CK7 (+), TTF‐1(+), vimentin (+), and CK20(−).

A left adrenal gland mass with obvious necrosis and remarkably increased FDG uptake was detected (SUVmax: 30.8; size: 7.6 × 5.1 × 5.0 cm), which was suspected to be metastases. Circumferential thickening of the small intestinal wall (up to 2.5 cm thickness) was found incidentally in the right middle abdomen (Fig 1), with intense FDG uptake (SUVmax: 41.6). The length of the involved intestine was about 4.5 cm. Based on these findings, we concluded that the patient had a possible intestinal metastasis of pulmonary sarcomatoid carcinoma. Subsequent pathology results from a CT‐guided biopsy confirmed adenocarcinoma, and immunohistochemical (IHC) staining results were as follows: cytokeratin (CK) (+); CK5/6 (−); P40 (−); thyroid transcription factor (TTF‐1) (+); programmed cell death 1 (PD‐1) (5%+); programmed cell death 1 ligand 1 (PD‐L1) (80%+); Ki‐67 (70%+); CK20 (−); CK7 (+); villin (−); and Satb2 (−). The surgical specimen of the intestinal mass following laparoscopic surgery showed poorly differentiated carcinoma with local sarcomatoid differentiation, infiltrating the whole layer with visible nerve and intravascular tumor thrombus, with IHC markers CK (+); CK7 (+); CK20 (−); TTF‐1 (+); vimentin (+); CD34 (vessel+); CD31 (vessel+); ETS related gene (ERG) (vessel+); CD117 (−); Dog‐1 (+); P63 (−); desmin (−); smooth muscle actin (SMA) (−); S‐100 (−); and Ki‐67 (70%+) (Fig 2). Taking the IHC markers into consideration, this patient was diagnosed as PSC with intestinal metastasis. He died four months after surgery.

### Case 2

Case 2 was a 61‐year‐old man whose main complaint was of melena of more than one month’s duration, and an intermittent fever up to 39.7°C for more than 20 days. He denied any symptoms of cough or abdominal pain, or other pulmonary or gastrointestinal discomfort. Gastrointestinal endoscopy at a local hospital had demonstrated an antral ulcer with normal intestinal mucosa. There was no improvement after administration of Yunnan white medicinal powder and colloidal bismuth pectin capsules. Initial laboratory tests indicated iron deficiency anemia and an albuminemic state with low hemoglobin (Hb) (73 g/L), total protein (51.1 g/L), albumin (27.5 g/L), prealbumin (39 mg/L), serum iron (2.3 umol/L) and total iron binding capacity (35.44 umol/L) level. The other laboratory results were normal, including electrolytes, blood sugar, tumor markers, hepatic and renal function.

**Figure 3:**
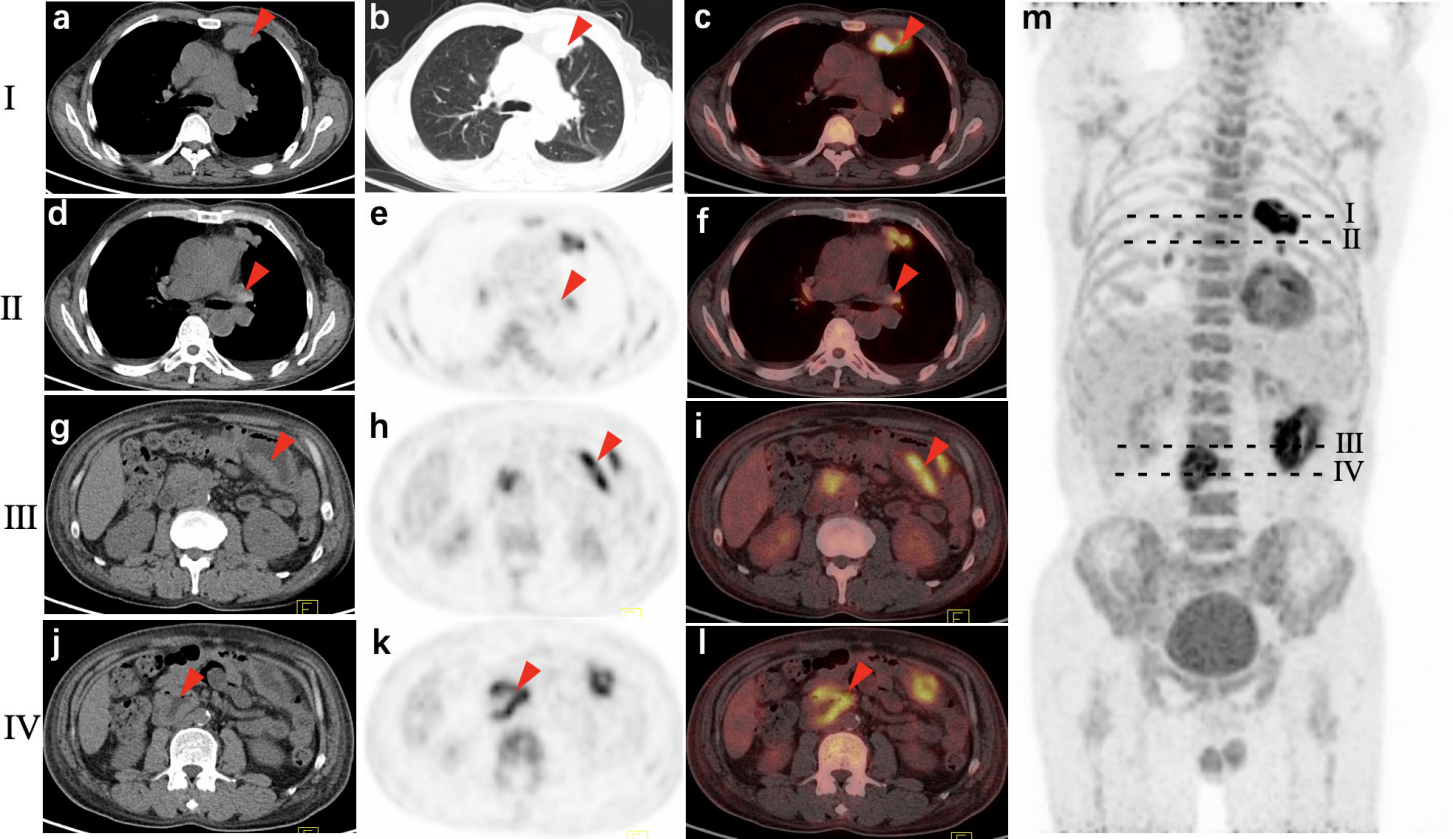
^18^F‐FDG PET/CT image of Case. **I**. (**a**) Soft tissue window; (**b**) lung window; and (**c**) PET/CT fusion image of left lung lesion manifested an irregular left upper lobe lung mass measuring 4.7 × 3.3 × 3.5 cm, with SUVmax of 13.4. The lesion showed malignant features including irregular margin, lobulation, spiculation, pleural traction, obvious necrosis of more than 30%, but without bronchial stricture and occlusion. ^18^F‐FDG PET/CT also detected wall‐thickening of descendant duodenum and part of jejunum in left upper quadrant of the peritoneal cavity with SUVmax of 10.4. The local intestinal cavity was dilated, and the surrounding fat gap was clear. **II**. (**d**) Transaxial CT; (**e**) PET; and (**f**) PET/CT fusion of bilateral hilar lymph nodes mildly enlarged with high density and symmetrically increased ^18^F‐FDG uptake (SUVmax: 4.0–5.3). **III** and **IV**. (**g**) Transaxial CT; (**h**) PET; (**i**) PET/CT fusion image of jejunum lesion; (**j**) transaxial CT; (**k**) PET; and (**l**) PET/CT fusion image of duodenum demonstrated thickening of the intestinal wall with intensive FDG uptake and dilatation of local intestinal cavity. (**m**) Maximum intensity projection image of whole body showed high uptake in lesions (red arrow) in left thorax (**I**); hilar lymph node (**II**); left middle upper abdomen (**III**); and right middle abdomen (**IV**).

^18^F‐FDG PET/CT showed an irregular large mass about 4.7 × 3.3 × 3.5 cm, with an SUVmax of 13.4 in the left upper lobe. The lesion showed malignant features including an irregular margin, lobulation, spiculation, pleural traction, and obvious necrosis of more than 30% without bronchial stricture and occlusion (Fig 3). The bilateral hilar lymph nodes were mildly enlarged with high density and symmetrically increased ^18^F‐FDG uptake (SUVmax: 4.0–5.3), and were considered as lymphadenitis. Meanwhile, ^18^F‐FDG PET/CT detected wall‐thickening of the descendant duodenum and part of the jejunum in the left upper quadrant of the peritoneal cavity with SUVmax of 10.4. The local intestinal cavity was dilated, and the surrounding fat gap was clear.

**Figure 4:**
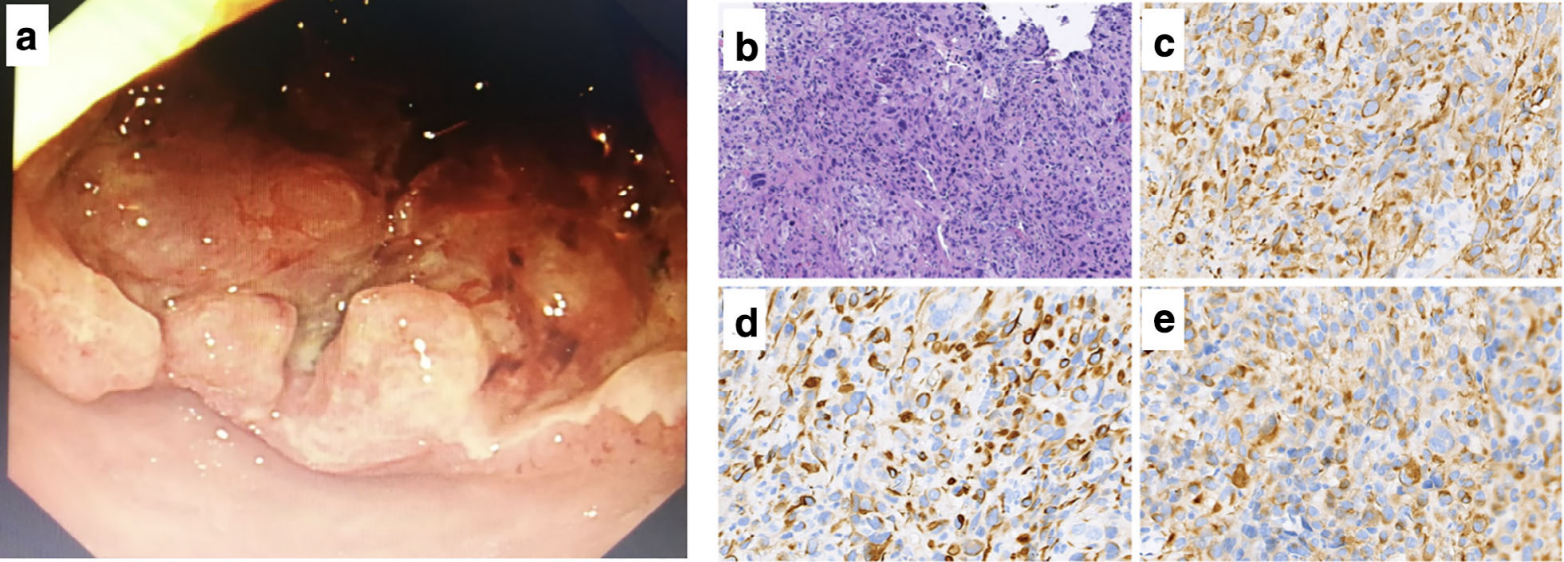
(**a**) Gastroscopic view; (**b**) HE staining; and (**c–e**) Immunohistochemical (IHC) staining of intestinal mass of Case (×200). (**a**) Gastroscopy demonstrated nodular eminence at the descending part of the duodenum with an obscure boundary, erosion and necrosis of the mucosal surface with dirty moss‐like appearance; (**b**) HE staining; and (**c–e**) IHC markers staining showed CK (AE1/AE3) (+); CK7 (+); and CK18(+).

Histopathological results from CT‐guided puncture of the pneumonic lesion suggested poorly differentiated NSCLC with massive necrosis, indicating PSC with IHC markers AE1/AE3 (+), vimentin (+), P40 (−), TTF‐1 (−), S‐100 (−), SMA (−), desmin (−), and Ki‐67 (20%+). Subsequent gastroscopy demonstrated nodular eminence at the descendant duodenum with an obscure boundary, erosion and necrosis of the mucosal surface with dirty moss‐like appearance (Fig 4a). Biopsy of the duodenum confirmed a poorly differentiated carcinoma with AE1/AE3 (+), CK7 (+), CK19 (−), CK8/18 (+), hepatocytes (−), epithelial membrane antigen (EMA) (+), TTF‐1 (−), and Ki‐67 (approximately 60%+) (Fig 4b), indicative of sarcomatoid carcinoma. The patient survived for five months after diagnosis.

## Discussion

PSC is rare, and gastrointestinal metastasis associated with the condition is exceedingly rare, with only 23 cases previously reported in the English‐language literature^5^, ^6^, ^7^, ^8^, ^9^, ^10^, ^11^, ^12^ None of the cases reported in the literature underwent ^18^F‐FDG PET/CT imaging. The detailed information on cases from included studies is listed in Table 1. The spread of PSC to the gastrointestinal tract occurs more frequently in the gastric and small intestinal region via hematogenous and lymphatic routes. Small bowel involvement regularly leads to perforation, obstruction and bleeding, resulting in shorter survival time in patients than those with other metastatic sites. In the current study, both patients had small intestinal metastasis and presented with symptoms of melena with a survival time of less than six months.

**Table 1:** Gastrointestinal metastases of pulmonary sarcomatoid carcinoma in 23 cases published in the literature since 1992

Author	Year	Case number	Symptoms	Sites	PET/CT scan performed (Yes/No)
Taira *et al*.^5^	2017	2	Abdominal pain, tarry stool	Small intestinal, gastric	No
Hu *et al*.^6^	2017	15	Hemorrhage, obstruction	Gastrointestinal tract	No
Chen *et al*.^7^	2015	1	Abdominal pain, poor appetite	Gastric and colonic	No
Romano *et al*.^8^	2015	1	Abdominal pain, vomiting	Small intestinal	No
Koh *et al*.^9^	2014	1	Abdominal pain	Small intestinal and gastric	No
Guner *et al*.^10^	2012	1	Abdominal pain, vomiting	Small intestinal	No
Kim *et al*.^11^	2008	1	Abdominal pain, melena	Small intestinal	No
Goh *et al*.^12^	2007	1	Hemorrhage	Gastric	No

Herein, we report ^18^F‐FDG PET/CT features of two cases of small intestinal metastases secondary to PSC. Both cases had initial abdominal symptoms whereas no pulmonary symptoms were present. The CT findings of the lung masses were not typical, with both cancerous and sarcomatoid features. Small intestinal metastases may not be related to obstruction and sometimes the local intestinal cavity may also be dilated. Nevertheless, the uptake of ^18^F‐FDG was remarkably high in both primary tumor and metastatic intestinal lesions, with SUVmax exceeding 30, similar to the study by Zhang *et al*. which reported a pulmonary sarcomatoid carcinoma with a SUVmax of 35.5.^13^ Rapicetta *et al*. also reported that PSCs often present with an intense uptake on ^18^F‐FDG PET/CT scan,^14^ which may correlate with high proliferation activity and high metastatic tendency. Remarkably high FDG uptake with bowel wall thickening or a mass may be helpful in the diagnosis of gastrointestinal metastases from PSCs. However, differential diagnosis from inflammatory lesions (such as diverticulitis), double cancer, and prominent physiological uptake are difficult.^15^

A diagnosis of sarcomatoid carcinoma is always dependent on IHC markers. With reference to ^18^F‐FDG PET/CT images, surgery or biopsy of a lung mass or intestinal lesion was performed, and sarcoma marker vimentin and carcinoma marker cytokeratin were finally found to be positive, which verified intestinal metastasis of PSC. The epithelial to mesenchymal transition pathway may play a key role. In both cases reported here, positive TTF‐1, CK and CK7 indicated lung origin, while negative CK20, CDX2 and villin excluded intestinal origin.

Early intervention can prevent patients with gastrointestinal metastasis of lung cancer from acute abdominal emergency, such as perforation and obstruction. Nishizawa *et al*. reported that the mean survival period of patients with gastrointestinal metastasis of lung cancer after bowel resection was 7.7 months, among which, one patient lived for 22 months.^16^ Enteral nutrition was restored as early as one month after surgery. Further, Kim *et al*. reported gastrointestinal metastasis in one case of PSC who achieved long‐term survival of more than five years after lung and intestinal surgery.^11^

In conclusion, for PSC patients with mild or without abdominal symptoms, 18F‐FDG PET/CT imaging could identify intestinal metastasis at a relatively early stage and may be used to determine the preferred biopsy site or early intervention by surgery.

## Disclosure

All the authors declare that they do not have any conflict of interest.
